# Archetypal Analysis Reveals Quantifiable Patterns of Visual Field Loss in Optic Neuritis

**DOI:** 10.1167/tvst.11.1.27

**Published:** 2022-01-19

**Authors:** Elena Solli, Hiten Doshi, Tobias Elze, Louis Pasquale, Michael Wall, Mark Kupersmith

**Affiliations:** 1 Deptartment of Neurology, Icahn School of Medicine at Mount Sinai, New York, NY, USA; 2 Albert Einstein College of Medicine, Montefiore Medical Center, Bronx, NY, USA; 3 Department of Ophthalmology, Icahn School of Medicine at Mount Sinai, New York, NY, USA; 4 Schepens Eye Research Institute, Harvard Medical School, Boston, MA, USA; 5 Departments of Neurology and Ophthalmology and Visual Sciences, University of Iowa, Iowa City, Iowa, USA

**Keywords:** archetypal analysis, visual field, optic neuritis, deep learning

## Abstract

**Purpose:**

Identifying and monitoring visual field (VF) defects due to optic neuritis (ON) relies on qualitative clinician interpretation. Archetypal analysis (AA), a form of unsupervised machine learning, is used to quantify VF defects in glaucoma. We hypothesized that AA can identify quantifiable, ON-specific patterns (as archetypes [ATs]) of VF loss that resemble known ON VF defects.

**Methods:**

We applied AA to a dataset of 3892 VFs prospectively collected from 456 eyes in the Optic Neuritis Treatment Trial (ONTT), and decomposed each VF into component ATs (total weight = 100%). AA of 568 VFs from 61 control eyes was used to define a minimum meaningful (≤7%) AT weight and weight change. We correlated baseline ON AT weights with global VF indices, visual acuity, and contrast sensitivity. For eyes with a dominant AT (weight ≥50%), we compared the ONTT VF classification with the AT pattern.

**Results:**

AA generated a set of 16 ATs containing patterns seen in the ONTT. These were distinct from control ATs. Baseline study eye VFs were decomposed into 2.9 ± 1.5 ATs. AT2, a global dysfunction pattern, had the highest mean weight at baseline (36%; 95% confidence interval, 33%–40%), and showed the strongest correlation with MD (r = −0.91; *P* < 0.001), visual acuity (r = 0.70; *P* < 0.001), and contrast sensitivity (r = −0.77; *P* < 0.001). Of 191 baseline VFs with a dominant AT, 81% matched the descriptive classifications.

**Conclusions:**

AA identifies and quantifies archetypal, ON-specific patterns of VF loss.

**Translational Relevance:**

AA is a quantitative, objective method for demonstrating and monitoring change in regional VF deficits in ON.

## Introduction

Optic neuropathies of all etiologies are associated with a broad spectrum of visual field (VF) defects, which are monitored using threshold perimetry. Idiopathic optic neuritis (ON), or ON associated with multiple sclerosis, frequently causes profound VF loss at presentation but recovers significantly over months, even without intervention. The clinical course of acute demyelinating ON is well-known owing to the carefully performed and widely disseminated results of the National Eye Institute–sponsored Optic Neuritis Treatment Trial (ONTT).^1^ The vast majority of eyes recover to have excellent visual acuity and normal mean deviation (MD) despite residual deficits in contrast sensitivity in 55.7% of affected eyes.^2^^–^^4^

The identification and monitoring of regional VF changes remains challenging. VF loss in ON typically shows specific spatial deficit patterns, but these patterns can vary widely among affected eyes. Common global indices of VF function such as MD quantify the extent of VF loss, but do not convey information regarding the various types of regional VF defects seen. Presently, accounting for the change in these patterns of dysfunction requires qualitative interpretation of repeat VFs. Small changes may not be recognized over time, particularly if there are more dominant patterns of VF loss present.^4^^–^^6^

Archetypal analysis (AA), a form of unsupervised machine learning, mathematically formalizes the qualitative approach discussed elsewhere in this article by determining representative patterns on the outer edges (the so-called convex hull) of the VF dataspace and can be used to extract and quantify archetypal component patterns from within a heterogeneous dataset without having any disease-specific background available.^7^^,^^8^ AA has previously been applied to VF analysis in glaucoma, another optic neuropathy, that, unlike ON, causes progressive VF deterioration over years. In glaucoma, AA reveals clinically relevant patterns of VF loss as archetypes (ATs) that can be quantified and monitored.^9^^–^^14^ These studies suggest that AA can be added to existing trend- or event-based VF indices, such as MD, to monitor VF changes in the assessment of optic neuropathies. Pattern standard deviation (PSD), total deviation (TD), and glaucoma hemifield changes are additional commonly used parameters of glaucomatous optic neuropathy VF deterioration, but to date there is little agreement that one method is superior for all stages of disease.^15^^,^^16^ Although the various algorithms are standardized, none are uniformly accepted and subtle pattern losses can be missed, may not track disease-specific deficits, are influenced by factors such as optical problems, and are often weighted to preferred regions, such as the central field. AA can reveal localized specific VF regions of interest not identified by global measures and can quantify specific patterns of VF loss similar to those described by experts, thus improving longitudinal analysis.^15^^,^^17^

In this study, we used AA to analyze VFs from a prospective, multisite treatment trial study of ON, the ONTT. We explored whether AA could produce ATs with clinically relevant patterns of VF deficits in acute ON, a disorder with a natural history course distinct from glaucoma. We hypothesized that AA could generate a model of quantitative, disease-specific defects (as ATs) in ON, and that the types of VF defects identified by this model would resemble known VF defects seen in ON.

## Methods

This study was approved by the Institutional Review Board of the Icahn School of Medicine at Mount Sinai. The ONTT study followed the tenets of the Declaration of Helsinki; informed consent was obtained from the subjects after explanation of the nature and possible consequences of the study. The ONTT was conducted under approval by the institutional human experimentation committee or institutional review board of each study site. A Data Safety and Monitoring Committee monitored the ethical conduct of the study and the accumulation of data.^1^

### Datasets

We performed AA on 3892 VFs, prospectively collected from 456 eyes during the ONTT, from acute presentation to the 1-year follow-up. Subjects with new-onset (within 8 days) acute ON in one eye were randomized to one of three treatment groups: placebo, three days intravenous methylprednisolone followed by 2 weeks of oral prednisone, or only oral prednisone for 17 days. A total of 151 eyes were treated with intravenous methylprednisolone, 156 with prednisone, and 149 with placebo. The mean age of all participants was 32 ± 6.7 years, and 77% were female.^1^ VF testing was performed by trained, certified technicians with quality control by an expert VF reading center.^6^ Two VF tests were done on each eye at study entry and at 6 months (trial outcome); otherwise, one VF was done on each eye for visits at 4, 15, 30, 49, 91, 133, 180, and 365 days. For 32 study eyes, no baseline VF had been performed owing to poor vision. For these eyes, a projected baseline VF was created: all raw sensitivity values were set to 0, from which TD values were derived. Reliability indices included fixation losses of less than 20% and false-positive and false-negative errors of less than 33%. Each study eye had the best-corrected visual acuity expressed as a logarithm of the minimum angle of resolution value and contrast sensitivity score of the number of characters identified using the Peli–Robson contrast charts. At baseline, the visual acuity was 0.2 (20/40) or better in 35.4%, between 0.40 and 0.98 (20/50–20/190) in 28.2%, and 1.0 (20/200) or worse in 36.3% of study eyes.^1^ At baseline, the mean visual acuity was 0.74 ± 0.66, the mean contrast sensitivity was 7.74 ± 4.83 letters, the MD was −21.52 ± 10.17 dB, and the mean PSD was 7.50 ± 4.01 dB (note that the PSD values only available for 338 eyes).

A separate dataset of 568 VFs, collected from 61 normal eyes from 61 subjects with 24-2 VF tests performed at multiple visits at the University of Iowa was used as a control group. The control VFs were used to create a normal AT model (for comparison with our ON AT model) and to determine the normal fluctuation in ON AT weights among healthy eyes between visits. The mean age of participants was 61.2 ± 8.9 years, and 63.3% were female. The normal participants met the following criteria: (1) no history of eye disease except refractive error (no more optical correction than 5 diopter of sphere or 3 diopter of cylinder), (2) no history of diabetes mellitus or systemic arterial hypertension, (3) a normal ophthalmologic examination, including 20/25 or better Snellen acuity, and (4) good fixation (losses og <20%) by gaze tracking or perimetrist observation, and (5) false-positive and false-negative rates of less than 10% on perimetry.^18^

### AT Analysis

**Figure 1.**
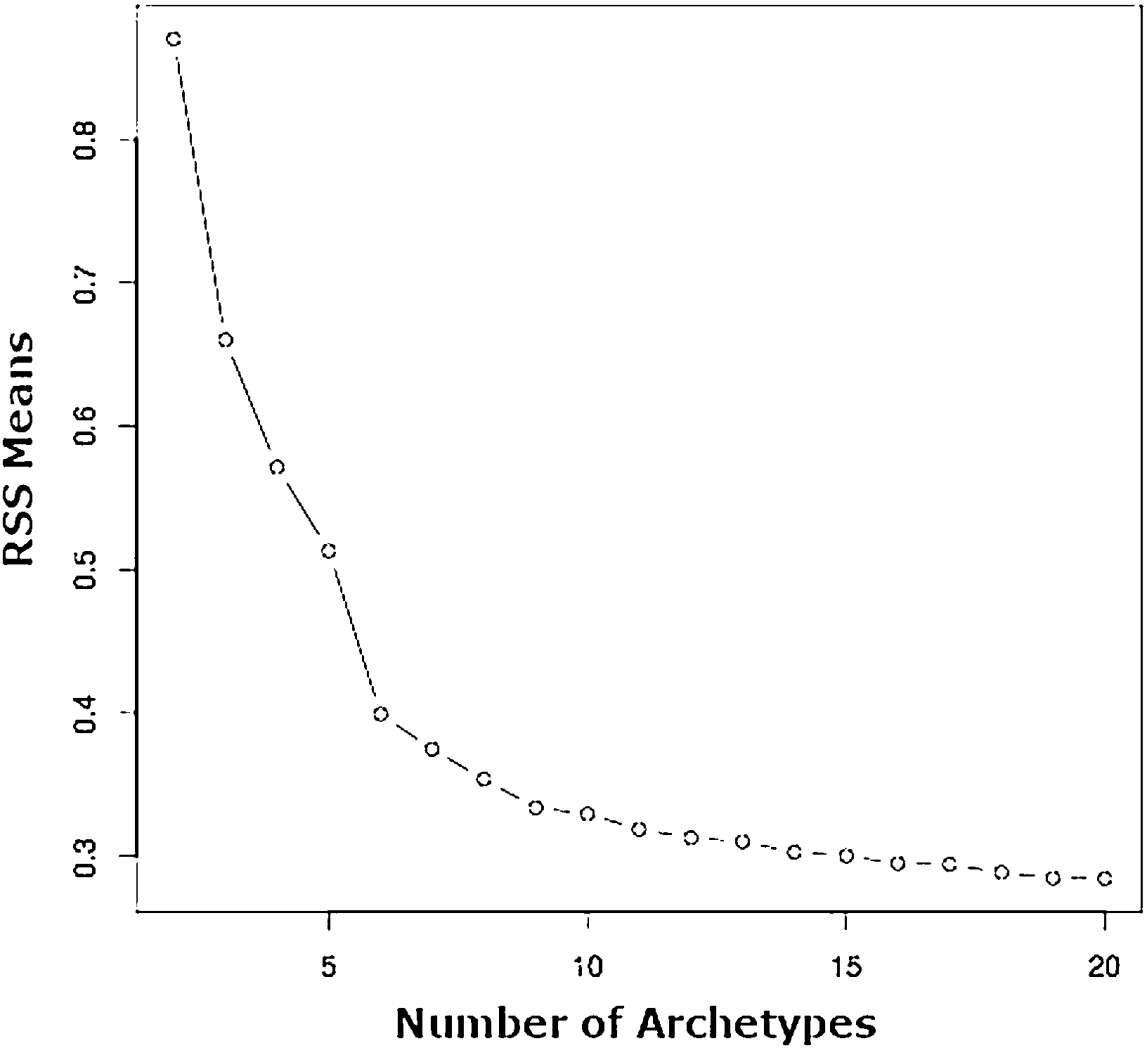
Residual sum of squares (RSS) plot generated during AA for the purposes of selecting the number of ATs. RSS values were normalized based on sample size. The final number of ATs for our model was selected based on the point at which this curve begins to flatten, to avoid overfitting; resulting in the selection of a 16-AT model.

To perform AA, we used the TD values extracted from our VF dataset as input data. Note that all VFs from visits up to 1 year were included as input data for AA. All left-eye VFs were converted to right-eye format, and all 30-2 VFs were converted to 24-2 before implementing AA. We used the open source software package “archetypes” within the R statistical programming environment (R Development Core Team 2008) to perform AA.^7^^,^^8^ To select the number of ATs for our model, we used 10-fold cross-validation for models using 2 to 20 ATs, such that all data were divided into 10 subsets, each of which was used as the testing set once, while the others served as the training set. We plotted the residual sum of the squares for each model as a function of the number of ATs, and selected a model from the flattened region of this curve, to avoid overfitting (Fig. 1). The sum of the relative weights (RW) of all ATs within this model was normalized to 100%. Using this model, each VF was decomposed into a set of ATs, each with its own RW, such that these RWs summed to 100%. For comparison, we used the same process to determine an AA model for the 61 control eye VFs. In both models, the ATs are numbered in order of RW, representing their frequency within the dataset, and each AT corresponds with an average TD value.

### Defining Threshold Value for Meaningful Weight and Weight Change

We defined a minimal meaningful weighting coefficient and weight change for ON ATs by first determining the weight and weight change of each ON AT among control eye VFs, tested repeatedly over 1 year. Weight changes that fall within the 95% confidence interval (CI) of this normal dataset represent normal fluctuations and are unlikely to represent a clinically important degree of change for determining optic nerve dysfunction. The highest upper limit of the 95% CI of mean weight change values for any abnormal ON AT among control eyes was 4.1% (95% CI 3.2%–4.1%) (Supplementary Table S1). We then considered the 16 AT model for the ONTT data (discussed elsewhere in this article), within which the weight of any individual AT would be equal to 1/16 (6.25%) if all AT weights were equal. We chose a conservative minimum weight value and change in weight of 7% or greater as meaningful for decomposed ATs for each study eye VF. Although smaller changes in AT weight have the potential to increase the sensitivity of change detection, in order to avoid decreased specificity we chose ±7% or greater as the threshold value for a meaningful weight change.

### VF Decomposition

We decomposed all study eye VFs into their component ATs using the 16 ON AT model (see Results), calculating the RW of all ATs for each individual VF at each time point. We determined the number of eyes with clinically relevant (meaningful) weight for each AT at each time point for all study eyes. We also calculated the cumulative mean weight for specific groupings of the more abnormal ATs, including the summed weights of two or more of the eight worst ATs at each time point.

### Comparison of ATs to Known ON VF Patterns

We categorized the 16 ON ATs patterns of VF defects and compared them with the expert classifications of the baseline VFs in the original ONTT.^5^^,^^6^ Classifications were available for a total of 360 baseline study eye VFs. We identified 191 baseline VFs that had a single dominant AT, defined as having an individual an AT of 50% or greater weighting.^9^ We compared the two groups of classifications, noting for each VF and dominant AT whether they matched exactly, partially (contained similar features), or not at all. Because the original expert classifications did not distinguish between superior and inferior VF defects or temporal and nasal VF defects, before matching analysis, the position of the VF defect was noted and taken into account during matching. The criteria for the matching analysis of descriptive patterns from expert and AT classifications are outlined in Supplementary Table S2.

### Statistical Analyses

All statistical analyses were performed using R software. We set the statistical significance level to α = 0.05. We used χ^2^ tests to evaluate for any significant difference between the treatment groups in terms of frequency of eyes with any AT weight of 7% or greater at baseline. We used Kruskal–Wallis tests to evaluate for any significant difference in AT weight values between the treatment groups at baseline (for any AT). We used Spearman's method for all correlation analyses and determined the correlation between each AT weight and MD, PSD, visual acuity (logarithm of the minimum angle of resolution), and Peli–Robson contrast sensitivity (number of letters seen). We also evaluated the correlation between PSD and the mean weight of ATs representing regional VF defects (these include all ATs except for AT2 and AT8, globally abnormal VFs, and AT1, a globally normal VF). We applied a Bonferroni correction to the resulting *P* values to account for multiple comparisons.

## Results

### The 16-AT Model for ON

**Figure 2.**
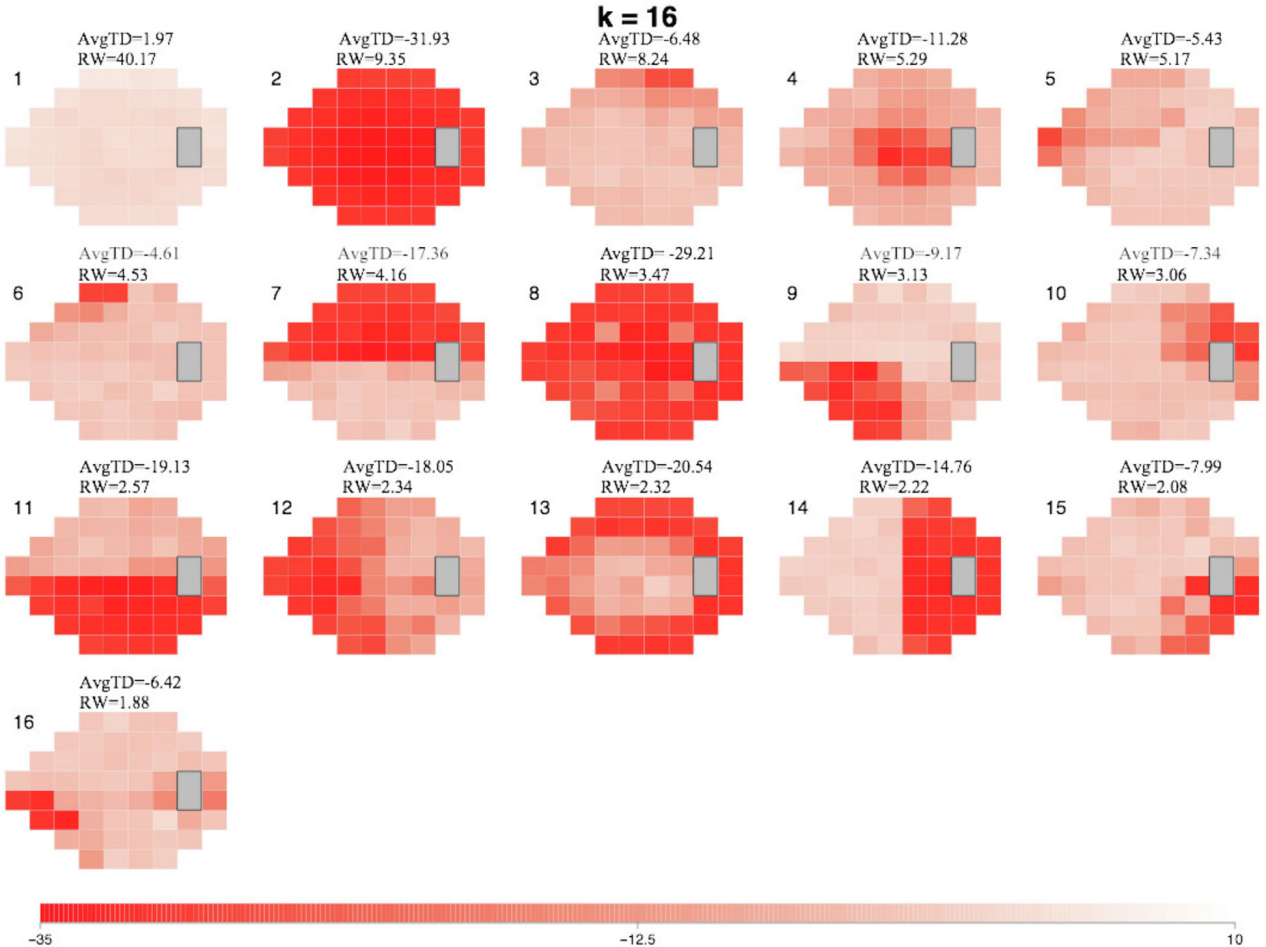
Map outlining the 16 ON-specific ATs contained within our model. The varying shades of red within each AT denote TD values, and scale at the bottom denotes the TD values associated with each shade. Each AT is shown along with its corresponding average TD value (avgTD) and RW within the dataset. The ATs are numbered and displayed in order of RW. Note the color scale range from –35 dB to 10 dB.

**Figure 3.**
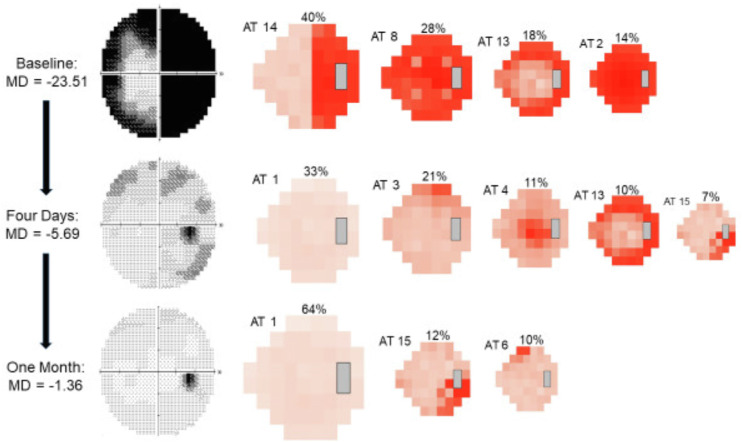
Example of VF decompositions from baseline to one month. The progressive changes in AT weighting and MD at each time point are displayed, along with the corresponding grayscale image from the Humphrey VF plot. AT weights not considered to be meaningful (< 7% at any time point) are not shown. Both MD and AT weights improved (weights of worse ATs decreased, weights of better ATs increased) starting at the 4-day visit after treatment with intravenous methylprednisolone.

The 16-AT model created from the ONTT VFs (Fig. 2) demonstrates a range of AT patterns, which are numbered in order of their RW (or frequency) within the entire dataset, at all time points. Each AT has an associated average TD value, which indicates its severity. AT1, representing a normal VF, had the highest RW at 40.17%, reflecting the significant recovery from ON that was observed in the ONTT. This was followed by AT2, representing diffuse VF loss, with a RW of 9.35%, reflecting the severe VF loss at presentation. An example of the decomposition of the VFs for one study eye from baseline to 1 month is illustrated in Figure 3. The decomposed ATs can reveal deficits not easily identifiable viewing the conventional VF. Although the gray scale of the actual 1-month VF shows no apparent abnormality, two of the three meaningful ATs show clear deficits, located in the superior nasal and inferior temporal regions.

A 12-AT model was the best fit for our dataset of control VFs (Supplementary Fig. S1) and showed no patterns typical of optic nerve dysfunction. These ATs were distinct from the ON ATs. The average TD value associated with each control AT was consistent with normal vision.

### Baseline AT Weights

**Figure 4.**
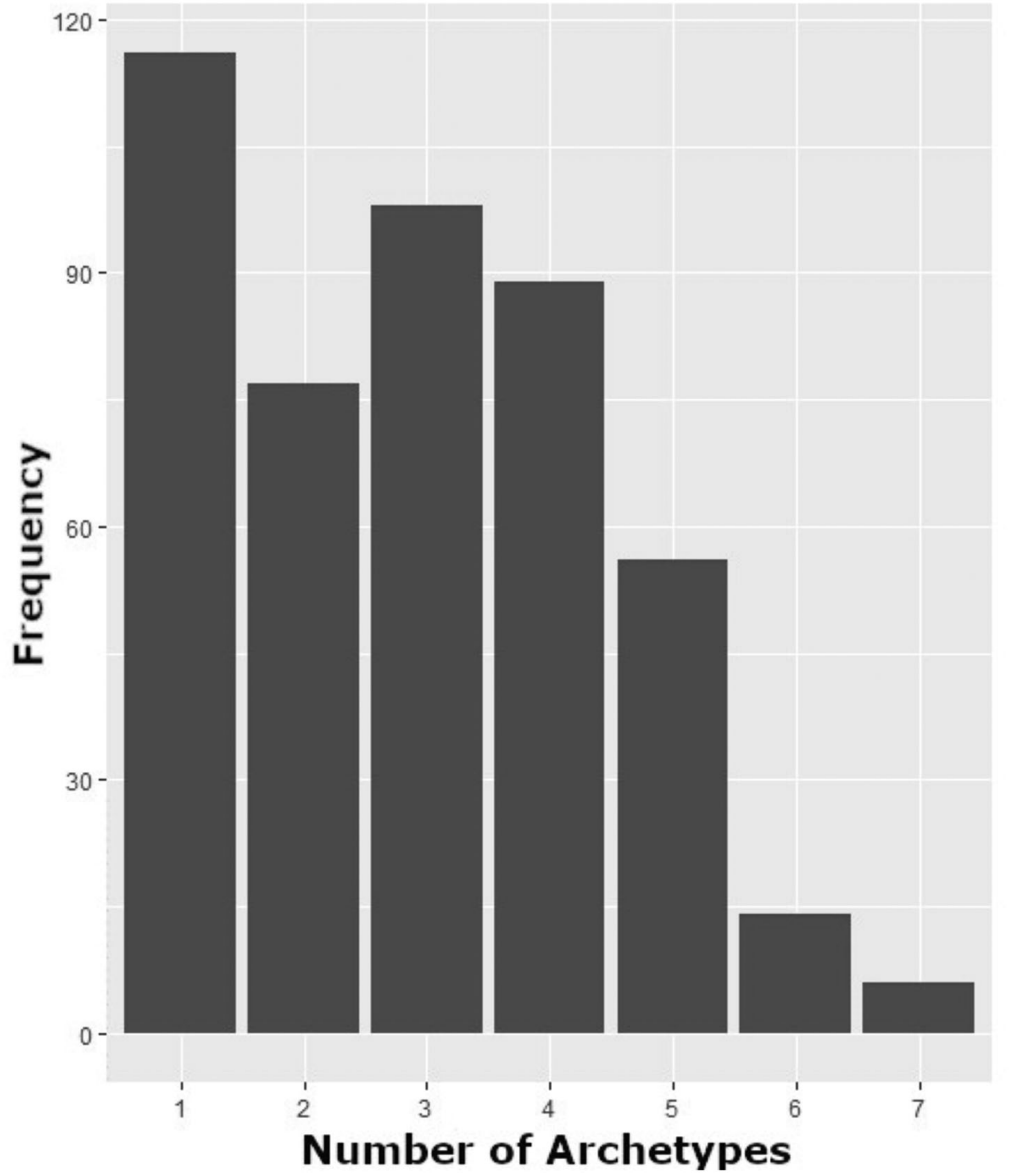
Frequency of baseline study eye VFs containing listed number of ATs of meaningful weight (≥ 7%).

**Figure 5.**
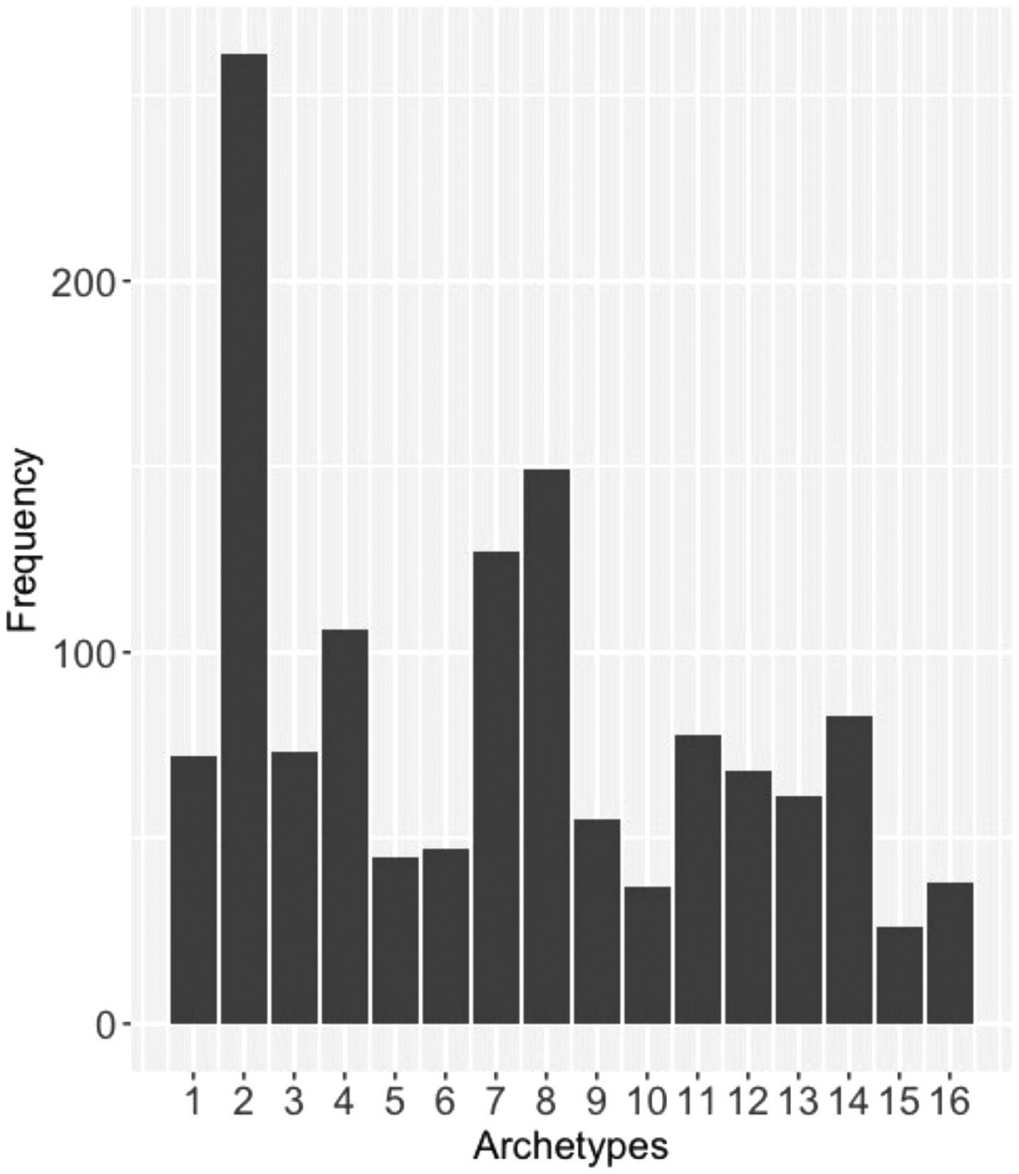
Frequency of study eyes with AT weight ≥7% at baseline, for each AT.

The baseline VFs of 436 eyes (96%) were decomposed into five or fewer meaningful ATs (mean 2.9 ± 1.5). No baseline VF for any study eye contained more than seven meaningful ATs (Fig. 4). AT2 had the highest frequency of study eyes, with meaningful weight at baseline (*N* = 261), followed by AT8 (*N* = 149), the second most severe AT (Fig. 5). AT1, representing a normal VF, had only 72 eyes with meaningful weight at baseline.

The distribution of the mean baseline AT weights for all study eyes is summarized in Supplemental Table S3. AT2 had the highest mean weight for any AT at baseline (36.4%; 95% CI, 0.33–0.40). The mean weight of AT1 at baseline was 5% (95% CI, 0.04–0.07). There was no significant difference between the treatment groups with regard to the frequency of eyes with meaningful weighting for any AT at baseline or the mean weights of any AT at baseline.

### Correlation of AT Weights with Other Measures of Vision Function at Baseline

**Figure 6.**
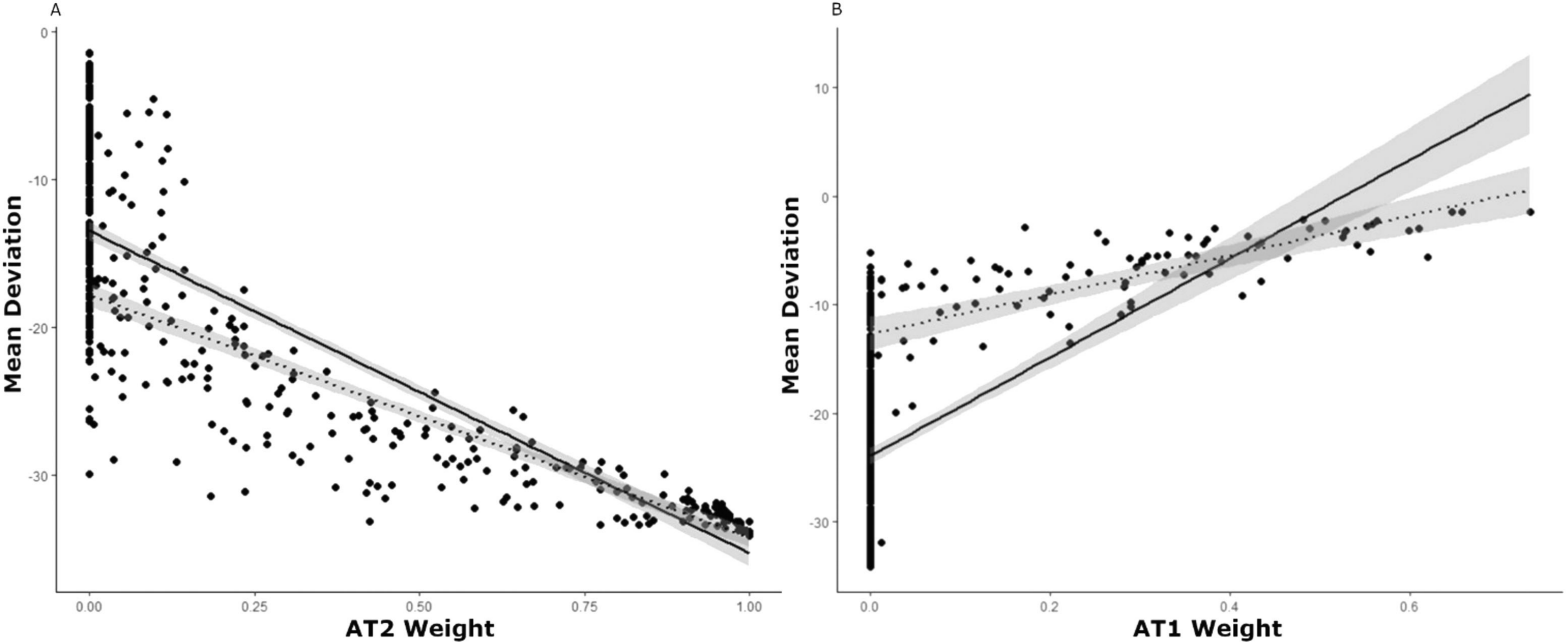
(A) Correlation between AT2 weight and MD (dB) at baseline (r = −0.91; *P* < 0.001), represented by the solid line. Dotted line represents the same correlation when eyes for which AT2 = 0% at baseline are eliminated (r = −0.94; *P* < 0.001). Note study eyes with an MD of <−20 dB that did not have high AT2 weight contained other ATs representative of severe VF loss. (B) Correlation between AT1 weight and MD (dB) at baseline (r = 0.63; *P* < 0.001). The trend line is skewed owing to the large number of study eyes with no AT1. The dotted line represents the same correlation when eyes for which AT1 = 0% at baseline are eliminated (r = 0.78; *P* < 0.001).

The AT2 weight had the strongest negative correlation with MD (r = −0.91; *P* < 0.001) (Fig. 6A) at baseline, while AT1 showed a moderate positive correlation (r = 0.63; *P* < 0.001) (Fig. 6B). Eliminating eyes that had an AT2 of 0% and an AT1 of 0% (respectively) at baseline (owing to the high level of data heteroscedasticity evident in these correlations), the correlation between AT2 and MD was unchanged (r = −0.94; *P* < 0.001), whereas the correlation between AT1 and MD increased (r = 0.78; *P* < 0.001). AT3 and AT6, both representing superior VF depression, were also moderately correlated with MD (AT3: r = 0.58; *P* < 0.001; AT6: r = 0.56; *P* < 0.001), as well as AT5 and AT16 (AT5: r = 0.50; *P* < 0.001; AT16: r = 0.49, *P* < 0.001), both representing nasal step-type patterns (Supplemental Table S4).

**Figure 7.**
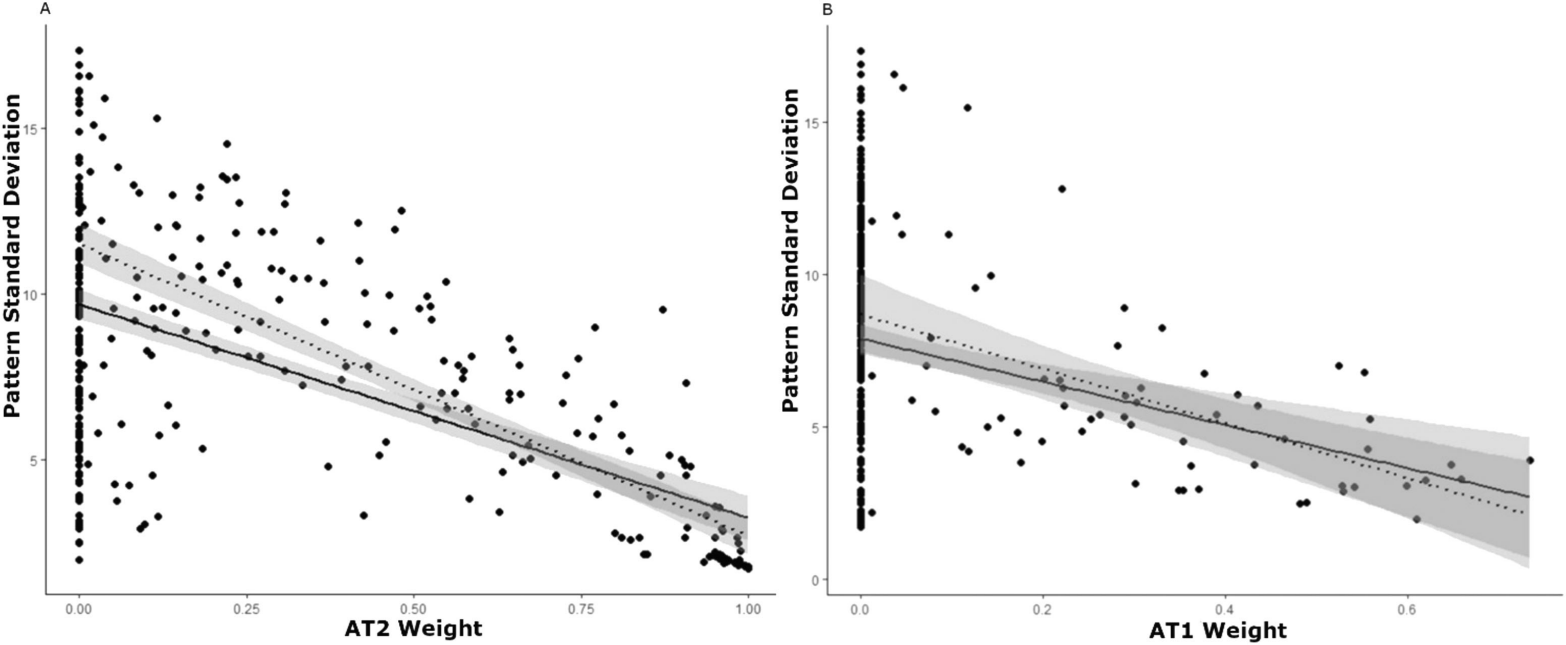
**(**A) Correlation between AT2 weight and PSD (dB) at baseline (r = −0.53; *P* < 0.001). The dotted line represents the same correlation when eyes for which AT2 = 0% at baseline are eliminated (r = −0.79; *P* < 0.001). Note the wide range of abnormal PSD values, when diffuse severe VF loss AT2 is 0%. (B) Correlation between AT1 weight and PSD (dB) at baseline (r = −0.2; *P* < 0.001). The dotted line represents the same correlation when eyes for which AT1 = 0% at baseline are eliminated (r = −0.49; *P* < 0.001). Note the wide range of abnormal PSD values, when the normal VF AT1 is 0%.

AT2 had a moderate negative correlation with PSD at baseline (r = −0.53; *P* < 0.001) (Fig. 7A), and AT1 was weakly negatively correlated (r = −0.20, p = 0.004) (Fig. 7B). Eliminating eyes that had an AT2 of 0% and an AT1 of 0% (respectively) at baseline, increased the correlation between AT2 and PSD (r = −0.79; *P* < 0.001), as well as the correlation between AT1 and PSD (r = −0.49; *P* < 0.001). Only two other ATs showed significant correlations with PSD at baseline (Supplemental Table S5). In addition, given that the PSD is lowest when a given VF is most uniform and higher when the VF is more irregular, we investigated the relationship between the PSD and the mean weight of only ATs defined as having primarily regional deficit (excluding AT1, AT2, and AT8), and found a strong correlation (r = 0.75; *P* < 0.001) (Supplementary Fig. S2).

For visual acuity at baseline, AT2 showed a strong positive correlation (r = 0.70; *P* < 0.001) (Supplementary Fig. S3)*.* AT1 and AT3 (AT1: r = −0.40; *P* < 0.001; AT3: r = −0.40; *P* < 0.001) showed modest negative correlations (note the use of the logarithm of the minimum angle of resolution scale; values increase with worse vision). For contrast sensitivity at baseline, AT2 showed the strongest negative correlation (r = −0.77; *P* < 0.001) (Supplementary Fig. S4)*.* AT1 showed a modest positive correlation (r = 0.46; *P* < 0.001), as well as AT3 (r = 0.44; *P* < 0.001), and AT6 (r = 0.41; *P* < 0.001). Almost all the other ATs showed a significant but weak correlation with the visual acuity and contrast sensitivity at baseline (Supplemental Tables S6 and S7).

### Comparison of ATs with Known ON VF Patterns

Previously categorized, descriptive patterns of VF defects seen at baseline included total loss, superior altitudinal, inferior arcuate, centrocecal, superior partial arcuate, inferior partial arcuate, inferior altitudinal, double arcuate, enlarged blind spot, temporal hemianopia, three-quadrant VF defect, multifocal VF defect, peripheral rim, central VF defect, and superior arcuate defect.^5^^,^^6^ Of the 191 VFs with a dominant AT at baseline, 133 (70%, which is 30% of the 456 total baseline VFs) showed a complete match between their descriptive classification and their dominant AT, whereas 21 (11%) showed a partial match (Table). Overall, 154 VFs (81%, which is 34% of the 456 total baseline VFs) showed at least a partial match between their previously classified VF defects, and their VF defects identified based on dominant AT (weight ≥50%).

**Table.** Matching Between ON ATs and Expert Classifications

AT	191 Study Eyes with a Dominant AT	Full Match, (*n* = 133)	Partial Match, (*n* = 21)	Full or Partial Match Total (*N* = 154)	Full Match	Partial Match
AT1	12	0	0	0	Within normal limits	N/A
AT2	111	109	0	109	Total loss	Cloverleaf, three-quadrant
AT3	4	0	4	4	Superior depression	Superior partial arcuate
AT4	5	3	0	3	Central, centrocecal, paracentral	Enlarged blind spot
AT5	4	0	2	2	Nasal step	Superior partial arcuate
AT6	0	0	0	0	Superior depression	Superior partial arcuate
AT7	18	9	0	9	Superior altitudinal	Superior arcuate, superior partial arcuate, double arcuate
AT8	9	0	9	9	Cloverleaf	Total Loss, Three-Quadrant
AT9	2	0	2	2	Inferior nasal quadrantanopia	Nasal step, nasal hemianopia, inferior altitudinal, inferior arcuate, inferior partial arcuate
AT10	0	0	0	0	Superior temporal quadrantanopia, temporal wedge	Temporal hemianopia, superior arcuate, superior partial arcuate
AT11	17	8	1	9	Inferior altitudinal	Inferior arcuate, inferior partial arcuate, double arcuate
AT12	3	0	2	2	Nasal hemianopia	Nasal step, superior nasal quadrant, inferior arcuate, inferior partial arcuate, three-quadrant
AT13	2	2	0	2	Peripheral rim or double arcuate	Superior arcuate, inferior arcuate
AT14	2	2	0	2	Temporal hemianopia	Temporal wedge, superior temporal quadrantanopia, inferior temporal quadrantanopia, three-quadrant
AT15	1	0	0	0	Inferior temporal quadrantanopia, temporal wedge	Temporal hemianopia, inferior partial arcuate
AT16	1	0	1	1	Nasal step	Inferior partial arcuate, enlarged blind spot

The number of study eyes at baseline with a dominant AT (≥50%) that had a full match or partial match with ONTT study expert classifications. Two right side columns show criteria used for matching.

## Discussion

Our results indicate that AA can identify disease-specific, archetypal patterns of VF loss in eyes with ON. These patterns are quantifiable and resemble known types of VF defects observed in the ONTT. AA is a form of unsupervised machine learning that can be used to analyze heterogeneous datasets. When applied to VFs, AA reveals identifiable representative patterns referred to as ATs within the dataset, such that any VF can be decomposed into a weighted sum of these patterns. AA has previously been applied to the monitoring of VF changes, disease progression and recognized expert-determined patterns in glaucoma.^9^^–^^14^^,^^19^ Recently, we showed that AA identifies recognizable patterns and uncovers regional deficits not seen by expert analyzers in VFs of eyes with papilledema due to idiopathic intracranial hypertension.^20^ With the exception of papilledema, AA has not yet been applied to the evaluation of VFs in nonglaucomatous optic neuropathies, where improvement, rather than deterioration, can occur with appropriate therapies. Our study extends the use of AA to the analysis of VFs in ON.

We created an archetypal model for ON by applying AA to a large dataset of VFs taken during the ONTT. For the majority of VFs with a dominant AT (≥50% weight) at baseline, the dominant AT matched the original descriptive classification for the VF from the original ONTT. This finding supports the assertion that this 16-AT model is specific to ON, and that AA identifies patterns of VF loss that are relevant clinically. In addition, in the original study, the majority of baseline VFs were characterized by diffuse loss (66.2% diffuse vs. 33.6% localized VF loss).^1^^,^^5^^,^^6^ As such, it is unsurprising that AT2, representing diffuse VF loss, was the most frequent abnormal AT (highest RW) within the entire dataset (which includes all time points) had the highest mean weight at baseline and represented more than one-half of the VFs with a dominant AT at baseline. Focal patterns observed in the ONTT included nerve fiber bundle abnormalities, such as arcuate and altitudinal defects, and in addition to central and centrocecal VF deficits. Partial arcuate, enlarged blind spot, vertical step, hemianopia, three-quadrant defects, paracentral loss, nasal steps, temporal wedge, quadrantanopia, double arcuate, peripheral rim, and superior depression were also noted.^5^^,^^6^ Nearly all of these types of VF defects were found among the 16 ATs.

As we anticipated, the 12-AT model from our dataset of normal control eyes did not overlap with the 16 ON ATs (except for ON AT1, a normal VF) and were consistent with normal vision. This contrast between the ON ATs and control ATs further suggests that AA is able to identify patterns that represent optic nerve dysfunction, and AA does not derive abnormal ATs from eyes with normal vision. Furthermore, by decomposing control VFs into ON ATs and examining how these weights changed over time, we quantified the extent to which AT weights normally fluctuate among healthy eyes. This process allowed us to define a conservative threshold value of 7% or greater to represent clinically relevant or meaningful weight change, distinguishable from normal AT weight fluctuation in eyes without VF defects. One limitation in this study was the use of control eyes from a population older than the ONTT study population. Because variability in VF testing increases with age, it is possible that the contrast between control ATs and ON ATs would have been greater had we been able to use younger age-matched controls. Also, the variability for patients without visual disease would certainly underestimate the variability for those with an acute severe vision loss from any disorder. Despite the limitation, we believe the selection of a conservative threshold for meaningful change in AT weight is supported further.

Of all ON ATs, AT2 showed the strongest negative correlation with MD, and AT1 showed the strongest positive correlation. This was expected, because AT2 represents total VF loss, and AT1 represents a normal VF; as VF function improves, the more normal ATs increase in weight and the worse ATs decrease in weight. Thus AT3, AT6, AT5, and AT16 were also moderately positive correlated with MD, because these ATs have relatively low average TD values compared with the other ATs.

Compared with its correlation with MD, we found a weaker correlation between AT2 and PSD. This outcome is likely because AT2 represents diffuse loss and is relatively uniform, and, as AT2 increases, the PSD should not increase as sharply. In contrast with its moderate correlation with MD, AT1 displayed a weak negative correlation with the PSD, possibly because as AT1 increases (as the VF normalizes), the PSD should decrease. In addition, we found a strong positive correlation between the PSD and the mean weight of all ATs representing regional defects (which by definition excluded AT1, AT2, and AT8). We anticipated this result, given that, as regional AT weights become more substantial, one would expect a corresponding increase in the PSD (owing to increased irregularity within the field). However, for each PSD value, the variance in the mean weight of the regional ATs may be considerable. Although MD indicates the overall severity of VF loss, the PSD is a pattern-based index, indicating the extent of variability within a VF.^21^ Commonly used VF global indices such as these may fail to convey fully the extent to which an individual VF has one or more regional patterns of VF loss that are seen in a specific disease (such as ON). One study of AA in glaucoma regarded AT1 (also their normal VF AT) weighting coefficients as a measure of how typical a given VF pattern is for glaucoma. Within this model, higher AT1 weights not only indicate that a VF is closer to normal, but also that the defect shown is less likely to be glaucomatous (if it were glaucomatous, there would likely be higher abnormal AT weights and a lower AT1 weight).^10^ Extending this concept to our study, in addition to the ON-specific abnormal ATs, the amount of AT1 weight may also specifically indicate the relevance of a given VF pattern to ON. AA seems to provide a new disease-specific, quantitative, pattern-based method of assessing the extent of various regional defects within a given VF, conveying information that global indices may fail to capture.

Notably, the correlation between AT1 and the MD improved substantially when eyes with AT1 weights of 0% were removed. This difference is likely because eyes with an AT1 weight of 0% represent eyes with regional patterns of VF loss (if AT1 is 0%, then the VF must be represented by the other ATs, which are abnormal). Thus, we observe that, when these eyes (those with regional VF loss, containing these abnormal ATs) are included in this correlation analysis, the correlation begins to break down, because the MD is agnostic to the specific type of regional VF deficit seen in a given field. When these eyes are removed, the correlation improves, because AT1 (a normal VF pattern) is essentially is the only AT remaining when disease patterns are removed. We also see that the correlation between AT2 and PSD as well as AT1 and PSD improve when eyes with AT1 and AT2 weights of 0% (respectively) are removed. This finding is due to the fact that essentially blind eyes and normal eyes are represented by PSD values close to zero; leading to a wide range of PSD values represented among eyes with an AT1 of 0% and an AT2 of 0%. The removal of eyes with AT1 and AT2 with 0% weight improves the correlation as these ends of the VF spectrum are eliminated. We suggest that, given the wide range of VF patterns seen in ON, as well as the fact that the MD and PSD are agnostic to both disease and pattern type, there is a need for new indices that quantify VF loss relative to disease-specific patterns.

## Conclusions

AA of VFs obtained from study eyes with acute unilateral vision loss during the largest clinical trial of ON successfully generated a set of clinically relevant, quantifiable, ON-specific patterns of VF loss. To date, the identification and monitoring of regional VF defects requires clinician interpretation and descriptive classification, which can vary among clinicians. AA quantifies these regional defects, which may facilitate the precise measurement and monitoring of VF changes without relying on qualitative interpretation and classification. AA may also reveal more subtle underlying VF defects that are otherwise difficult to detect, which enhances the detection of regional defects over time. Further research will include testing of the ON-specific ATs to determine the effects of therapies and in real-world datasets of patients who have had ON. AA should advance structure–function investigations by quantifying residual AT abnormalities to correlate with optical coherence tomography measurements of regional retinal nerve fiber layer and macula ganglion cell layer thinning.

## Supplementary Material

Supplement 1
