# Supplementary material for: Archetypal Analysis Reveals Quantifiable Patterns of Visual Field Loss in Optic Neuritis
Source: Transl Vis Sci Technol. 2022 Jan 19;11(1):27. doi: 10.1167/tvst.11.1.27 (PMC8787544; doi:10.1167/tvst.11.1.27)
Supplement: Supplement 1 [file tvst-11-1-27_s001.docx]

**SUPPLEMENTAL FIGURES AND TABLES:**

| **Archetype** | **Mean Change** | **95% CI Lower Bound** | **95% CI Upper Bound** |
| --- | --- | --- | --- |
| AT1 | .831 | .822 | .840 |
| AT2 | .003 | .002 | .004 |
| AT3 | .036 | .032 | .041 |
| AT4 | .005 | .004 | .006 |
| AT5 | .025 | .022 | .028 |
| AT6 | .034 | .031 | .038 |
| AT7 | .005 | .004 | .006 |
| AT8 | .0004 | .00006 | .0008 |
| AT9 | .011 | .009 | .012 |
| AT10 | .011 | .009 | .012 |
| AT11 | .004 | .003 | .005 |
| AT12 | .004 | .003 | .006 |
| AT13 | .007 | .006 | .009 |
| AT14 | .005 | .005 | .006 |
| AT15 | .011 | .009 | .012 |
| AT16 | .006 | .005 | .006 |

**Supplemental Table 1:ON AT weight fluctuation among normal (control) eyes.** Mean weight change (with 95% CI) of control VFs decomposed into ON ATs (averaged over all time points).


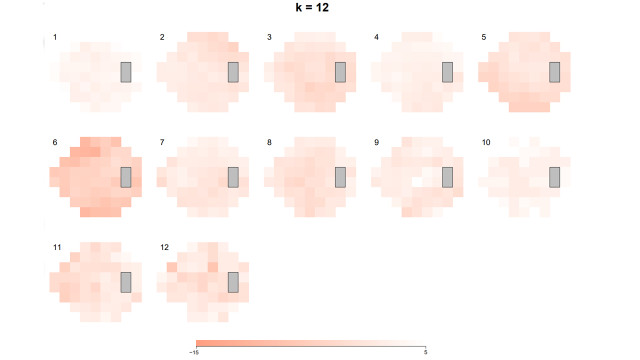


**Supplemental Figure 1:**Map of 12 control ATs, derived from dataset of VFs taken from normal, healthy eyes. Note the color scale ranges from -15 dB to 5 dB, as the control eyes have minor minus total deviations.

| **Archetype** | **Mean Weight at Baseline** | **95% CI Lower Bound** | **95% CI Upper Bound** |
| --- | --- | --- | --- |
| AT1 | .053 | .040 | .066 |
| AT2 | .364 | .327 | .402 |
| AT3 | .041 | .031 | .051 |
| AT4 | .052 | .042 | .063 |
| AT5 | .024 | .017 | .032 |
| AT6 | .020 | .015 | .026 |
| AT7 | .100 | .082 | .118 |
| AT8 | .087 | .072 | .101 |
| AT9 | .031 | .022 | .039 |
| AT10 | .018 | .013 | .023 |
| AT11 | .070 | .059 | .087 |
| AT12 | .039 | .029 | .048 |
| AT13 | .027 | .020 | .034 |
| AT14 | .040 | .031 | .049 |
| AT15 | .014 | .009 | .019 |
| AT16 | .019 | .013 | .025 |

**Supplemental Table 3:Baseline AT Weight Coefficient Distribution for all ATs (**for all study eyes).

| **Archetype** | **r** | **P-value** |
| --- | --- | --- |
| AT1 | 0.627 | < 0.001 |
| AT2 | -0.914 | < 0.001 |
| AT3 | 0.581 | < 0.001 |
| AT4 | 0.477 | < 0.001 |
| AT5 | 0.504 | < 0.001 |
| AT6 | 0.558 | < 0.001 |
| AT7 | 0.376 | < 0.001 |
| AT9 | 0.308 | < 0.001 |
| AT10 | 0.332 | < 0.001 |
| AT15 | -0.139 | 0.047 |
| AT16 | 0.488 | < 0.001 |

**Supplemental Table 4:Mean Deviation and AT Weight Correlation.**Spearman correlation between each AT weight and mean deviation at baseline. Correlations for AT8, AT11, AT12, and AT13 are not shown as these were not statistically significant.

| **Archetype** | **r** | **P-value** |
| --- | --- | --- |
| AT1 | -0.199 | 0.004 |
| AT2 | -0.531 | < 0.001 |
| AT7 | 0.400 | < 0.001 |
| AT15 | -0.312 | < 0.001 |

**Supplemental Table 5:Pattern Standard Deviation and AT Weight Correlation.** Spearman correlation between each AT weight and pattern standard deviation at baseline (only ATs with statistically significant correlations shown).


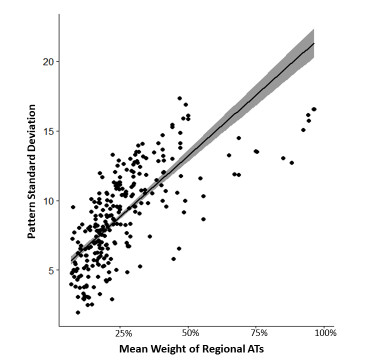


**Supplemental Figure 2:** Correlation between pattern standard deviation (db) and the mean weight of all regional ATs: those defined by a primarily regional deficit, including all ATs except AT1, AT2, and AT8 (r = 0.75, p < 0.001).


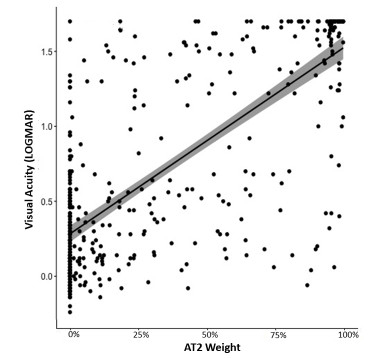


**Supplemental Figure 3:** Correlation between AT2 weight and logMAR visual acuity at baseline (r = 0.7, p < 0.001).


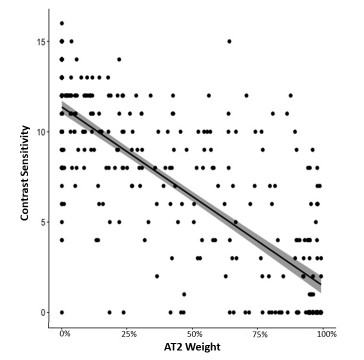


**Supplemental Figure 4:** Correlation between AT2 weight and contrast sensitivity (letters seen) at baseline (r = 0.77, p < 0.001).

| **Archetype** | **r** | **P-value** |
| --- | --- | --- |
| AT1 | -0.403 | < 0.001 |
| AT2 | 0.700 | < 0.001 |
| AT3 | -0.401 | < 0.001 |
| AT4 | -0.149 | 0.023 |
| AT5 | -0.327 | < 0.001 |
| AT6 | -0.356 | < 0.001 |
| AT7 | -0.285 | < 0.001 |
| AT9 | -0.328 | < 0.001 |
| AT10 | -0.154 | 0.015 |
| AT13 | -0.193 | < 0.001 |
| AT15 | 0.191 | < 0.001 |
| AT16 | -0.305 | < 0.001 |

**Supplemental Table 6:Visual Acuity and AT Weight Correlation.** Spearman correlation between each AT and visual acuity (logMAR) at baseline. Correlation coefficients are displayed with their corresponding p-values (only statistically significant correlations shown).

| **Archetype** | **r** | **P-value** |
| --- | --- | --- |
| AT1 | 0.455 | < 0.001 |
| AT2 | -0.771 | < 0.001 |
| AT3 | 0.436 | < 0.001 |
| AT4 | 0.165 | 0.006 |
| AT5 | 0.354 | < 0.001 |
| AT6 | 0.407 | < 0.001 |
| AT7 | 0.321 | < 0.001 |
| AT9 | 0.283 | < 0.001 |
| AT10 | 0.193 | < 0.001 |
| AT13 | 0.204 | < 0.001 |
| AT15 | -0.174 | 0.003 |
| AT16 | 0.364 | < 0.001 |

**Supplemental Table 7:Contrast Sensitivity and AT Weight Correlation.** Spearman correlation between each AT and contrast sensitivity at baseline. Correlation coefficients are displayed with their corresponding p-values (only statistically significant correlations shown).
